# Effectiveness of internet-delivered psychological treatments for children and adolescents with anxiety and/or depressive disorders: Systematic review and network meta-analysis

**DOI:** 10.1016/j.ijchp.2024.100487

**Published:** 2024-07-18

**Authors:** Concepción López-Soler, Jose Luis Vicente-Escudero, Jose Antonio López-López, Mavi Alcántara, Antonia Martínez, Maravillas Castro, Visitación Fernández, Julio Sánchez-Meca

**Affiliations:** a Department of Personality, Evaluation and Psychological Treatment, University of Murcia, Espinardo Campus, 31, Murcia, Spain; b Department of Basic Psychology and Methodology, University of Murcia, Espinardo Campus, 31, Murcia Spain

**Keywords:** Internet psychological treatments, Children and adolescents, Network meta-analysis, Anxiety, Depression

## Abstract

**Background:**

Anxiety and depression symptomatology has increased in the child and adolescent population. Internet-delivered psychological treatments (IDPT) can help to reduce this symptomatology, attending to the largest possible population.

**Aim:**

To conduct a systematic review and network meta-analysis of IDPT to reduce anxiety and depression symptoms in children and adolescents.

**Methods:**

The search for studies was conducted in SCOPUS, PsycINFO, PSICODOC, PsycARTICLES and Medline, between 2000 and 2022, in December 2022. Studies were selected if they were conducted with a sample of children and/or adolescents with previous symptoms of anxiety and depression, had applied IDPT, and included at least two comparative groups with pretest-posttest measures. Network meta-analyses were separately performed for anxiety and depression outcomes. Publication bias was analyzed using Egger's test and funnel plots, and mixed-effects meta-regression models were applied to account for heterogeneity.

**Results:**

37 studies were included in the meta-analysis, providing a total of 74 comparative groups. IDPT exhibited low-to-moderate, statistically significant average effect sizes when compared to both inactive and active controls. No statistical significance was found when IDPT was compared with other types of interventions.

**Discussion:**

IDPT is recommended to reduce anxiety and depression symptomatology in children and adolescents, but more studies are needed which compare treatments with other types of interventions, such as face-to-face therapy.

## Introduction

In recent years, several epidemiological studies have noted an increase in internalizing symptomatology among adolescents. In a sample of the general population in the US for instance, rates of 31 % of clinically significant anxiety and 14.3 % of depression were found among adolescents (Merikangas et al., 2010). Other studies have indicated that the worldwide prevalence of anxiety disorders in children and adolescents in the general population is 6.5 % and 2.6 % in depression (Polanczyk et al., 2015). Moreover, an increase in prevalence of anxiety and depression has been observed in the general population of children and adolescents of up to 26 % in anxiety and 29 % in depression following the COVID-19 pandemic (Ma et al., 2021).

The increase in internalizing psychopathology is related to exposure to traumatic and stressful experiences such as confinement. These variables are related to a high presence of various psychological disorders, greater functional deterioration and duration of symptoms (Briere et al., 2008; Castro et al., 2019; Hodges et al., 2013).

As shown in meta-analyses, the exposure of children and adolescents to one or more of these risk situations is linked to the development of several psychopathologies, including internalizing symptoms like anxiety and depression (Humphreys et al., 2020), externalizing ones such as antisocial behaviors (Degli et al., 2020), or subsequent substance use (Yoon et al., 2020). However, leisure activities, social, parental or school support, or emotional intelligence often act as protective factors (Cadamuro et al., 2021; Crescentini et al., 2020; Flores et al., 2020; Mostan et al., 2021).

Given the high prevalence of internalizing problems among children and adolescents, a crucial goal is to develop psychological care strategies which can reach large numbers of children and adolescents. In this vein, internet-delivered psychological treatments (IDPT) are financially viable, and can be administered to a large population, especially when users cannot travel to their therapy centers (Mitchell et al., 2021). IDPT are defined as those treatments designed to provide psychological support through different online platforms, such as emails, text messages, real-time chats, discussion forums, pre-recorded videos, live video sessions, serious games, videoconferencing or any other type of platform where participants can interact with the web and access psychological therapy from their homes, using devices such as computers, smartphones or tablets (Cuijpers et al., 2015; Mora et al., 2008).

New technologies are often portrayed as a source of addiction and internalizing or externalizing problems in adolescents (Vicente-Escudero et al., 2019). Nonetheless, when used as a platform to deliver psychological therapies, they can exert a positive influence (Heber et al., 2017), offering advantages over conventional (e.g., face-to-face) therapy such as time flexibility, anonymity or financial profitability (Sander et al., 2016).

IDPTs have proliferated in recent years (Wozney et al., 2017), and in some countries are already considered second-line psychological treatments when first-line treatments are ineffective or cannot be delivered (Renton et al., 2014).

Some meta-analyses have found significant reductions in anxiety and depression symptomatology after applying online psychological therapies in children and adolescents (Buttazzoni et al., 2021; Karyotaki et al., 2018; Valimaki et al., 2017). However, many systematic reviews and meta-analyses agree that the biggest problems when analyzing the effectiveness of these treatments lies in the small number of articles of high methodological quality (Ye et al., 2014), the great diversity of treatment programs developed, and in the heterogeneity of study samples (Rooksby et al., 2015).

### Objectives

The main purpose of this research was to investigate the effectiveness of IDPT to ameliorate symptomatology in children and adolescents with depressive and/or anxiety disorders, both for clinical and subclinical populations. We were also interested in estimating the effectiveness of IDPT using different comparison groups, namely active and inactive control groups as well as other active psychological interventions. Lastly, we also aimed to identify participant and treatment moderators of the effectiveness of IDPT. Furthermore, we used network meta-analysis (NMA) for statistical integration in order to more efficiently compare the diversity of intervention programs developed.

## Method

This investigation was a systematic review and network meta-analysis that adhered to PRISMA-NMA checklist (Hutton et al., 2015) to correctly report this type of research (see Table s4 in Supplementary file).

### Selection criteria

Eligibility criteria for primary studies were defined using the PICOS tool as follows (Higgins et al., 2019). (a) *Participants*: the study had to include samples of children and/or adolescents with depressive and/or anxiety disorders, accepting both clinical and subclinical populations. Participants with an official diagnosis (DSM-5 or ICD-11) of anxiety and/or depression were considered clinical population and participants without an official diagnosis, but who passed screening cut-off points to be included in the study, were considered subclinical population. (b) *Interventions*: the study had to apply any IDPT aimed to ameliorate depressive and/or anxiety symptoms. (c) *Comparison group*: one or more comparison groups, including inactive (e.g., waiting list) or active (e.g., a psychological placebo) control groups, as well as other psychological interventions. (d) *Outcomes*: the study needed to measure depressive and/or anxiety symptoms (both of them primary outcomes of this review) with validated measurement scales. (e) *Study design*: randomized and non-randomized multiple groups designs were accepted, with at least pretest-posttest measures. Additional selection criteria were: (f) published or carried out between 2000 and 2022, (g) written in English or Spanish, and (h) reporting statistical data for calculating the effect sizes (means, SDs, sample sizes for pretest and posttest of each group).

### Study search

Several search strategies were employed to locate studies. Firstly, the electronic databases SCOPUS, PsycINFO, PSICODOC, PsycARTICLES, and Medline were consulted in December 2022, covering years 2000–2022. The following keywords were combined: [“Internet based intervention” OR “Internet intervention” OR “Web based intervention” OR “Web intervention” OR “Online based intervention” OR “Online intervention” OR “Multimedia based intervention” OR “Multimedia intervention” OR “Internet based treatment” OR “Internet treatment” OR “Web based treatment” OR “Web treatment” OR “Online based treatment” OR “Online treatment” OR “Multimedia based treatment” OR “Multimedia treatment” OR “Internet based therapy” OR “Internet therapy” OR “Web based therapy” OR “Web therapy” OR “Online based therapy” OR “Online therapy” OR “Multimedia based therapy” OR “Multimedia therapy”] AND [Child* OR Adolescen* OR Teen* OR Youth OR Young]. Second, references from published meta-analyses and systematic reviews on a similar topic were reviewed (Arnberg et al., 2014; Brigden et al., 2020; Buhrman et al., 2016; Büscher et al., 2020; Buttazzoni et al., 2021; Clarke et al., 2015; Ennis et al., 2018; Fleming et al., 2014; Karyotaki et al., 2018; McGar et al., 2019; Reis et al., 2021; Renton et al., 2014; Reyes-Portillo et al., 2014; Rice et al., 2014; Rooksby et al., 2015; Santilhano, 2019; Siemer et al., 2011; Stasiak et al., 2016; Valimaki et al., 2017; Wozney et al., 2017; Ye et al., 2014). Finally, references of studies located and included were reviewed. The flow diagram in Supplementary Figures 1 describes the literature search process.

This combination of search strategies enabled us to select 37 studies conducted between 2009 and 2022. However, three studies were excluded for various reasons. Two did not report statistical data for calculating effect sizes and no answer was received to a request for additional information (Donovan et al., 2017; Sapru et al., 2016). A third study was excluded (Hearn et al., 2018) as its sample of participants coincided with that from other study included in the meta-analysis (Spence et al., 2017). Therefore, this meta-analysis contained a total of 34 studies (33 published studies and one unpublished doctoral thesis) (Fleming et al., 2012; Gladstone et al., 2018; Hetrick et al., 2017; Hill & Pettit, 2016; Hoek et al., 2011, 2012; Ip et al., 2016; Jolstedt et al., 2018; Karbasi & Haratian, 2018; Lindqvist et al., 2020; Makarushka, 2012; March et al., 2009; Merry et al., 2012; Moeini et al., 2019; Nordh et al., 2021; Poppelaars et al., 2016; Rickhi et al., 2015; Schleider et al., 2022; Schniering et al., 2022; Smith et al., 2015; Spence et al., 2011, 2017; Stasiak et al., 2014; Stjerneklar et al., 2019; Tillfors et al., 2011; Topooco et al., 2018, 2019; Topper et al., 2017; Vigerland et al., 2016; Waite et al., 2019; Waters et al., 2015, 2016; Wright et al., 2017; Wuthrich et al., 2012).

### Data extraction

Study characteristics were systematically extracted through a prior protocol. In particular, methodological, participant, treatment, and context characteristics were extracted from the studies. To assess methodological quality of studies, several items from the PEDro scale (Verhagen et al., 1998) and Cochrane risk of bias checklist (Higgins et al., 2019) were adapted to produce a 9-item checklist: (1) eligibility criteria were specified, (2) participants were randomly allocated to groups, (3) random allocation was concealed, (4) groups were similar at baseline regarding most important prognostic indicators, (5) there was blinding of all assessors who measured at least one key outcome, (6) measures of depression and anxiety outcomes were obtained from over 85 % of participants initially allocated to groups, (7) all participants for whom outcome measures were available received treatment or control condition as allocated or, when this was not the case, data for depression and anxiety outcomes were analyzed by “intention to treat”, (8) absence of within-study reporting bias (the study provided both point measures and measures of variability for all outcomes described in the Methods section), and (9) depression and anxiety outcomes were assessed with validated measurement scales.

Regarding participant characteristics, the following variables were extracted: psychological disorder of sample (depression, anxiety, or both), depressive symptoms (major depression, suicide thoughts, self-harm), anxiety disorders (generalized anxiety, social phobia, separation anxiety, panic disorder, agoraphobia, posttraumatic stress disorder), target population (clinical vs. subclinical), sample age (mean, standard deviation, range), gender distribution (% female), socioeconomic status (low, medium, high), with whom the child/adolescent resided (both parents, only one parent, other family members), whether medication was stabilized during the trial, and education level of mother and father.

As for treatment characteristics, the following variables were extracted: theoretical model (cognitive-behavioral therapy, social cognitive theory, psychodynamic models), focus of treatment (only the children/adolescents vs. both children/adolescents and parents), whether treatment was manualized, cognitive-behavioral techniques (psychoeducation, emotion regulation and acknowledging, problem solving, cognitive restructuring, behavioral activation, gradual exposure, relaxation, social skills), homework, therapist involvement (low, medium, high), parental involvement, treatment intensity (hours per week), treatment duration (number of weeks), and treatment quantity (total number of hours). Cognitive-behavioral techniques were coded as present only if explicitly mentioned in the treatment description; otherwise, these were coded as absent. Additionally, several contextual characteristics were extracted: publication year of study, country (and continent) of study, and location where families resided (urban vs. rural).

To assess reliability of data extraction process, two coders independently extracted the characteristics of all studies by applying the prior protocol. Inconsistencies were resolved by consensus. Supplementary file includes inter-rater agreement coefficients (Cohen's kappas and intraclass correlations) reached in the reliability analysis (Table s1). The methodological quality assessment of studies was also subjected to reliability analysis by two independent coders. The supplementary file includes results (Cohen's kappas) of these analyses (Table s2). As shown in these tables, all kappa coefficients and intraclass correlations were satisfactory (over 0.7).

### Effect size index

Each study might include two or more groups (e.g., IDPT vs active control; IDTP, active control, and inactive control), all with pretest and posttest measures. For each group, a standardized mean change index was calculated, *g*, this defined as the difference between the average pretest-posttest change divided by the pretest standard deviation, *S_Pre_*. This index was corrected with Hedges’ correction factor, *c*(*m*) = 1 – 3/(4*n* – 5), with *n* being the sample size. Thus, the effect size formula was (Morris, 2008):

$$g=c\left(m\right)\frac{{\bar{y}}_{Pre}-\;{\bar{y}}_{Post}}{S_{Pre}}$$

Positive *g* indices indicated a decrease in symptoms from pretest to posttest. The sampling variance of *g, V*(*g*) was calculated by means of (Morris, 2008):

$$V\left(g\right)=\;c{\left(m\right)}^{2}\left[\frac{2\left(1-r\right)}{n}\right]\left(\frac{n-1}{n-3}\right)\left[1+\frac{ng^{2}}{2\left(1-r\right)}\right]-g^{2}$$

In this formula, *r* was the Pearson correlation coefficient between pretest and posttest scores. As *r* is not commonly reported in the studies, following Rosenthal's (1991) recommendation, it was imputed as *r* = 0.7. In case of attrition, *g* indices were calculated with the means and standard deviations reported for intent-to-treat analyses.

From each study and group, a *g* index was calculated separately for depressive and anxiety symptoms. When a study reported several measurement tools for depression or anxiety, we selected one following as a rule child/adolescent self-reports over parent or clinician reports. If a study reported several self-report outcomes for depression or anxiety, we selected the measurement tool used most frequently in the rest of the papers included in our study, in order to homogenize the measurement instruments as much as possible.

In order to compare effects among different treatment and control groups in the same study, a *d* index was calculated, defined as the difference between the two *g* indices: *d* = *g*_1_ – *g*_2_, where labels 1 and 2 refer to two groups in the study (e.g., IDTP vs. active control). The sampling variance of the *d* index was calculated as the sum of variances of the two *g* indices: *V*(*d*) = *V*(*g*_1_) + *V*(*g*_2_). Thus, a study with only two groups (e.g., IDPT vs. active control) allowed calculating only one *d* index. A study with three groups (e.g., IDPT, active control, and inactive control) allowed calculating three *d* indices: IDPT vs. Active control, IDPT vs. Inactive control, and Active control vs. Inactive control. All these *d* indices were used as the dependent variable in the meta-analytic models (see next section). It is important to note that the effect size index used in this meta-analysis takes into account the pretest measurements in order to control for potential differences between the two groups in the pretest. This effect size is not the one usually applied in meta-analyses on intervention efficacy, which typically use only posttest mean differences. This circumstance can limit the comparability of our results with those of previous similar meta-analyses. However, our effect size is the most appropriate for controlling internal validity threats when comparing two independent groups on a continuous variable with both pretest and posttest measures (Morris, 2008). In any case, to the extent that the two groups are balanced in the pretest, the two types of effect sizes will converge.

### Statistical synthesis

Separate network meta-analyses (NMAs) were conducted, one for depression and another for anxiety outcomes. As a first step, network plots were created to examine the network geometry for each outcome (Dias et al., 2013). Treatment nodes in the plot are proportional to the number of children and adolescents randomized to each intervention, whereas lines connecting treatments represent direct evidence (e.g., head-to-head comparisons reported in primary studies), with line thickness proportional to the number of children contributing to each treatment comparison. A random-effects NMA model using restricted maximum likelihood estimation was then performed for each outcome. In the statistical analyses each effect size was weighted by its inverse variance, the latter defined as the sum of within-study and between-study variances.

In each NMA, treatments examined in at least 20 patients were included in the main network for each outcome, and the main parameters to be estimated for interpretation were the average treatment effects between each pair of interventions (represented as *d_+_*). Inconsistency was assessed for each outcome by fitting a model first proposed by Jackson et al. (2016), but using likelihood-based estimation methods as presented in Law et al. (2016). This model provides estimates the $\tau_{\beta }^{2}$ and $\tau_{\omega }^{2}$ variance components, which quantify residual heterogeneity and inconsistency, respectively. Profile likelihood confidence intervals (CIs) of both parameters were also obtained.

In order to explain heterogeneity, mixed-effects meta-regression models were applied taking effect sizes as the dependent variable and characteristics extracted from the studies as potential predictors, such as sociodemographic and clinical characteristics of participant samples and specific techniques implemented in the cognitive-behavioral treatments (e.g., psychoeducation, emotion regulation, problem solving, cognitive restructuring, behavioral activation, relaxation, gradual exposure). The statistical significance of each moderator was assessed with the improved *F*-test developed by Knapp and Hartung (2003; Viechtbauer et al., 2015). The proportion of variance explained by each moderator was estimated by means of the *R*^2^ index (López-López et al., 2014). Lastly, funnel plots and regression tests for funnel plot asymmetry (Egger et al., 1997) were performed to assess potential publication bias for each outcome.

Data were initially stored in a database using IBM SPSS, and then read into R for statistical analyses (R Core Team, 2022), using the metafor (Viechtbauer, 2010) and network packages (Butts, 2008).

## Results

### Study characteristics

Of the 34 studies included, twenty-nine studies comprised two groups, four studies three groups, and one study had four groups. In total, 74 groups were included in this meta-analysis: 38 applied some kind of IDPT, 32 were control groups (19 active and 13 inactive control groups), 3 applied face-to-face psychological treatments, and one applied a hybrid treatment (online and face-to-face). Studies were carried out in Europe (14 studies: seven in Sweden, three in The Netherlands, three in the UK, and one in Denmark), Oceania (11 studies: eight in Australia and three in New Zealand), North America (six studies: five in the USA and one in Canada), and Asia (three studies: two in Iran and one in Hong Kong). The total sample of participants was 5689, with a median of 40 participants per group (mean = 77; minimum = 9; maximum = 821; *SD* = 157).

The main characteristics of samples of participants are shown in Table 1, Table 2. The mean age of samples was 14.3 years old overall (range = 8.5 – 17.7), with a larger presence of females than males (average percentage of females = 72.6 %; range = 29.4 % - 100 %), living with both parents (average percentage = 71 %), and mainly in urban areas (average percentage = 77.6 %). Thirty-eight of the 74 groups comprised clinical samples (51.4 %), and 34 groups of subclinical participants (45.9 %), two groups consisted of a mixture of both clinical and subclinical participants (2.7 %). From 74 groups, 39 had a depression disorder (or depressive symptoms) without anxiety (52.7 %), 28 had an anxiety disorder (or anxiety symptoms) without depression, and seven groups had both depressive and anxiety symptoms (9.5 %).

**Table 1:** Continuous characteristics of the studies.

Study characteristic	*N*	Min.	Max.	Mean	*Md*	*SD*
Sample characteristics: Average age (years) SD of the age (years) Gender (% female) Setting (% in urban zone) Living with both parents (%) Target population (% clinical) Depressive symptoms (%) Severe depression (%) Stabilized medication (%) Other psychological treatment (%)	71 67 72 25 30 74 65 59 35 42	8.5 0.6 29.4 0 26.0 0 0 0 0 0	17.7 2.8 100 100 96.1 100 100 100 34.6 92.3	14.3 1.5 72.6 77.6 71 53.4 61.6 9.4 4.3 4.7	14.8 1.4 69.7 100 74.7 100 100 0 0 0	2.4 0.5 17.0 33.4 18.5 49.7 45.7 25.8 7.3 19.8
Treatment characteristics: Treatment intensity (nº hours per week) Treatment length (nº weeks) Total treatment (total nª of hours)	47 48 48	0.3 1 1	3.5 15 24	0.9 8.2 7.6	1 8 5	0.6 3.4 5.5

*N* = number of groups. Min. and Max. = Minimum and Maximum values. *Md* = Median. *SD* = Standard deviation.

**Table 2:** Categorical characteristics of the studies.

Characteristics of the samples	*N*	%
Target population: Clinical Subclinical Mixed Type of disorder: Depression Anxiety Both depression and anxiety	38 34 2 39 28 7	51.4 45.9 2.7 52.7 37.8 9.5
Characteristics of the treatments	*N*	%
Type of treatment: IDPT Face-to-face treatment Mixed treatment Active control group Inactive control group Theoretical treatment model: CBT Psychodynamic Social cognitive theory CBT with spiritual principles Any mental health delivery Search for positive stimuli Information delivery Improving children-parent relationships None Treatment focus: Children/adolescents Parents Both Manualized treatment: Yes No Therapist involvement: Null Low High Parent involvement: Null 1 activity >1 activity	43 5 1 5 20 37 1 1 2 2 2 6 1 22 40 1 11 46 5 16 21 14 35 1 14	58.1 6.8 1.4 6.8 27.0 50.0 1.4 1.4 2.7 2.7 2.7 8.1 1.4 29.7 76.9 1.9 21.2 90.2 9.8 31.4 41.2 27.3 70.0 2.0 28.0

Table 1, Table 2 also present treatment characteristics of studies. Forty-three of the 74 groups applied any type of IDPT (58.1 %), five groups applied face-to-face psychological treatments (6.8 %), one received both internet and face-to-face treatment (1.4 %), five were active control groups (6.8 %), and 20 were inactive control groups (27 %). Regarding the theoretical psychological model applied, the most represented was CBT with 37 groups (50 %). Other psychological approaches were minority, such as psychodynamic treatment (one group, 1.4 %), social cognitive theory (1 group, 1.4 %), CBT with spiritual principles (two groups, 2.7 %), any mental health delivery system (two groups, 2.7 %), search for positive stimuli (two groups, 2.7 %), information delivery (six groups, 8.1 %), and improving children-parent relationships (one group, 1.4 %). From 43 IDPT groups, 40 focused on children/adolescents (76.9 %), 11 groups in both the children/adolescents and parents (21.2 %), and one group focused on parents (1.9 %). Most IDPT groups applied a manualized treatment protocol (90.2 %). Therapist involvement in treatment was null in 31.4 % of groups, low in 41.2 %, and high in 27.3 %. Parental involvement was null in 70 % of treatment groups, low in 2 %, and high in 28 % (Table 2).

Table 1 shows other treatment characteristics. Treatment intensity, defined as number of hours of treatment per week ranged from 0.3 to 3.5 h per week (median = 1 hour). Treatment length, defined as number of weeks of treatment, ranged between 1 and 15 weeks (median = 8 weeks), and total hours of treatment received by participants ranged from 1 to 24 h (median = 5 h).

Table 3 presents the specific CBT techniques applied in the different studies. The most common technique was psychoeducation (93.9 %), followed by emotional acknowledgement and cognitive restructuring (73.5 %), problem solving (57.1 %), relaxation (55.1 %), emotional regulation (53.1 %), behavior activation (38.8 %), social skills (36.7 %), and gradual exposure (26.5 %). Homework was applied in 59.2 % of treatments.

**Table 3:** Treatment techniques included in the CBT interventions.

Treatment technique	*N*	%
Psychoeducation Emotion acknowledgement Emotional regulation Problem solving Cognitive restructuring Behavior activation Gradual exposure Relaxation Social skills Homework	46 36 26 28 36 19 13 27 18 29	93.9 73.5 53.1 57.1 73.5 38.8 26.5 55.1 36.7 59.2

The methodological quality of studies was assessed with nine items selected from the PEDro scale and Cochrane's risk of bias items. Table 4 presents compliance with each quality item. Total compliance of the studies was achieved for items 1 (sample eligibility criteria) and 9 (use of validated measurement tools), followed by items 2 (random allocation) and 8 (absence of reporting bias), with 97.1 % of compliance. Items 4 (equivalence of groups in pretest) and 7 (use of intent-to-treat analysis) obtained 85.3 % of compliance. The three items with low compliance were item 5 (blinding of assessors), with 41.2 % of compliance, item 3 (allocation concealment), with 64.7 %, and item 6 (less than 15 % of attrition between pretest and posttest), with 67.6 %. The supplementary file includes a table with individual compliance of each of the 34 studies (Table s3).

**Table 4:** Compliance with the methodological quality items (*k* = 34).

Quality item	Compliance (%)
1. Sample eligibility criteria 2. Random allocation 3. Allocation concealment 4. Pretest equivalent groups 5. Blinding of assessors 6. Less than 15 % of attrition 7. Intent to treat analysis 8. Absence of reporting bias 9. Validated measurement tools	100 97.1 64.7 85.3 41.2 67.6 82.4 97.1 100

### Network meta-analyses

Separate NMAs were conducted for depression and anxiety symptoms. Treatment categories included IDPT, Face-to-face Therapy, and Hybrid Therapy for CBT interventions, whereas other theoretical approaches were kept separately.

**Fig. 1:**
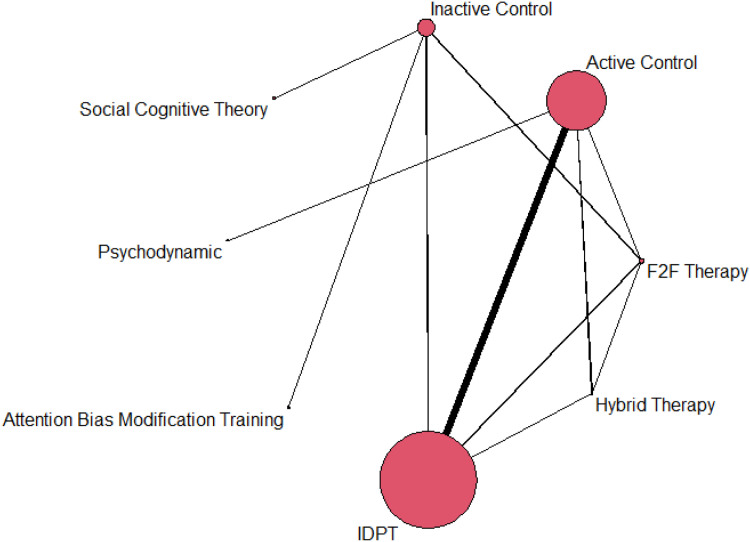
Network plot for depression.

**Fig. 2:**
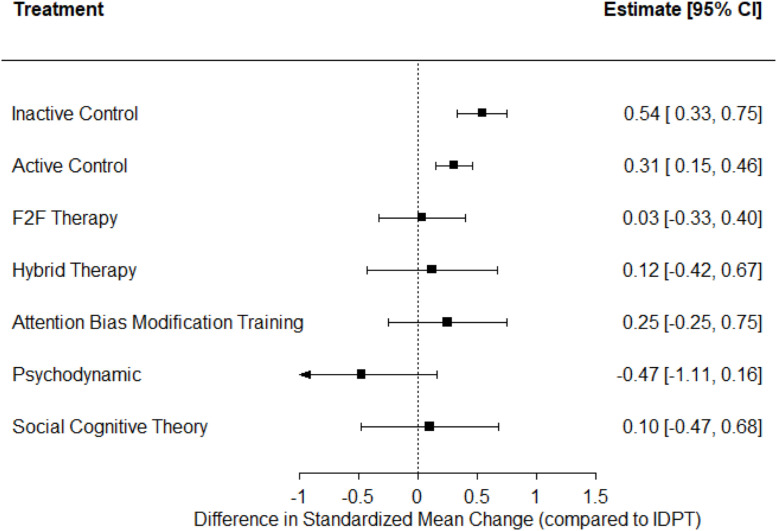
Forest plot with NMA results for depression. Positive values indicate that the reference treatment (IDPT) yielded a larger decrease in anxiety. F2F = face-to-face.

Fig. 1 presents a network graph with results for depression, and Fig. 2 shows a forest plot with the average effect sizes comparing IDPT with each of the alternative comparators. As shown in Fig. 1, IDPT vs active control was the most represented comparison, followed by IDPT vs inactive control. IDPT obtained the largest effect size when compared with inactive and active control groups (*d*_+_ = 0.54 and 0.31, respectively), reaching statistical significance in both cases. When IDPT was compared with the remaining comparators, a non-statistically significant average effect size was found: ‘Attention Bias Modification Training’ (*d*_+_ = 0.25), ‘Hybrid Therapy’ (*d*_+_ = 0.12), ‘Social Cognitive Theory’ (*d*_+_ = 0.10), and ‘Face-to-face Therapy’ (*d*_+_ = 0.03). When IDPT was compared with a group that applied psychodynamic therapy, the effect size favored the latter (*d* = −0.47), although not reaching statistical significance.

The estimate of $\tau_{\beta }^{2}$ was 0.05, with a profile likelihood CI between 0.01 and 0.14, which suggests the existence of residual heterogeneity. In addition, $\tau_{\omega }^{2}$ was estimated at 0, hence the standard NMA (assuming consistency) and the inconsistency NMA model yielded identical results.

**Fig. 3:**
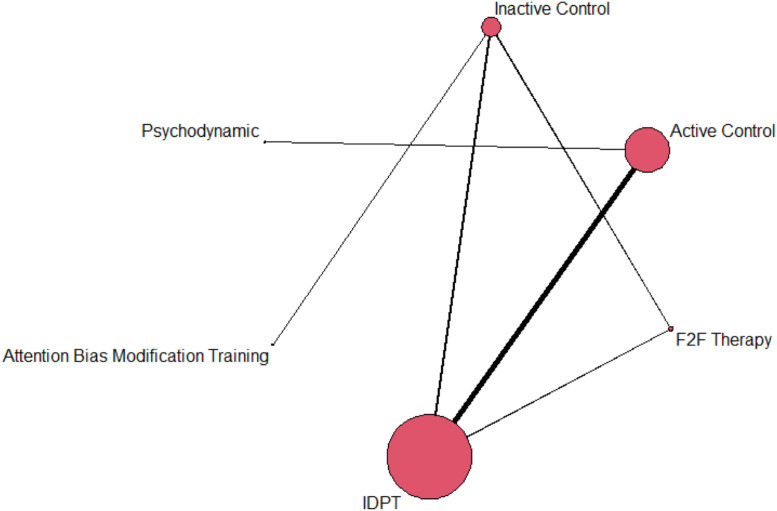
Network plot for anxiety.

**Fig. 4:**
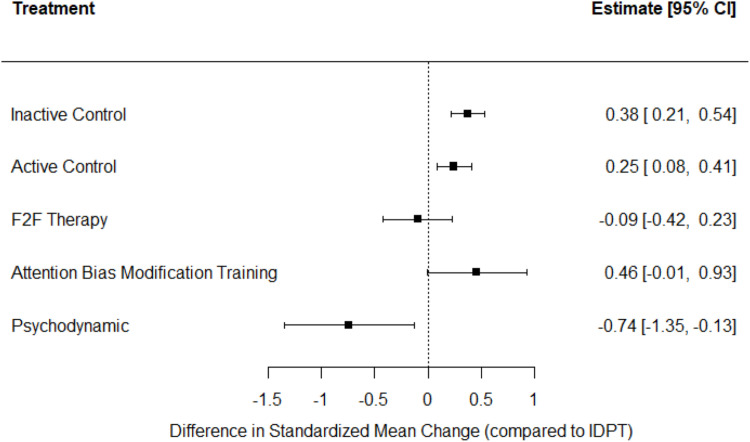
Forest plot with NMA results for anxiety. Positive values indicate that the reference treatment (IDPT) yielded a larger decrease in anxiety. F2F = face-to-face.

As regards anxiety, Fig. 3, Fig. 4 present a network graph and forest plot, respectively, illustrating results for this outcome. As in the case of depression, IDPT vs active control was the most represented comparison, followed by IDPT vs inactive control. IDPT exhibited a statistically significant average effect size when compared with inactive and active control groups (*d*_+_ = 0.38 and 0.25, respectively), but not when compared with ‘Attention Bias Modification Training’ (*d*_+_ = 0.46). IDPT obtained a negative average effect size when compared with ‘Face-to-face Therapy’ (*d*_+_ = −0.09) and one group that applied psychodynamic therapy (*d* = −0.74), reaching statistical significance in this last case.

The estimate of $\tau_{\beta }^{2}$ was 0.04, with a profile likelihood CI between 0.01 and 0.12, which suggests the existence of residual heterogeneity. Moreover, $\tau_{\omega }^{2}$ was estimated at 0, and hence the standard NMA (assuming consistency) and the inconsistency NMA model yielded identical results.

### Additional analyses

The existence of residual heterogeneity in both depression and anxiety NMAs, led us to search for study characteristics that might explain at least part of the effect size heterogeneity. The vast majority of comparisons were between IDPT and control groups (inactive and active controls), so that the search for moderators of heterogeneity focused on IDPT-control comparisons only. As a result, there was only one effect parameter to estimate (i.e., the effect of IDPT compared to a control group), and hence the network meta-regression model collapsed to a mixed-effects meta-regression model where the influence of each moderator was assessed by means of the expression: $y_{AB}=\beta_{0}+\beta_{1}X_{1}+\beta_{2}X_{2}$, with $y_{AB}$ being the effect size that compared IDPT with a control group (active or inactive), *X*_1_ representing a dummy variable for the type of control group (0: inactive; 1: active), and *X*_2_ the moderator to be tested. The moderators were tested one at a time, controlling for type of comparator and using the untruncated correction of Knapp and Hartung. Publication bias was also assessed separately for each comparison (e.g., IDPT vs. active control and IDPT vs. inactive control).

The results of the meta-regressions for effect sizes on depression are shown in Table 5. Once the influence of type of control group was controlled (active vs. inactive), none of the moderators exhibited a statistically significant relationship with the effect sizes (*p* > .05). Funnel plots – presented in Figure s2 – showed notable asymmetry compatible with publication bias for IDPT vs. active control (*p* = .02), but not for IDPT vs. inactive control (*p* = .79).

**Table 5:** Results of the mixed-effects meta-regression models applied on effect sizes for depression (*k* = 22).

Moderator	${\hat{\beta }}_{2}$	95 % CI for ${\hat{\beta }}_{2}$	*p*	$\Delta R^{2}$
% Clinical Population	0.001	−0.003, 0.003	.925	0 %
Depressive	0.227	−0.092, 0.625	.137	9.08 %
Anxious	−0.163	−0.581, 0.255	.425	0
Focus	−0.029	−0.414, 0.356	.878	0
Emotion Count	0.041	−0.331, 0.412	.921	0
Emotion Regulation	−0.155	−0.428, 0.117	.249	5.90 %
Problem Solving	−0.246	−0.494, 0.002	.052	15.7 %
Cognitive Restructuring	0.112	−0.296, 0.519	.574	0
Behavioural Activation	0.171	−0.113, 0.455	.223	0
Gradual Exposure	−0.125	−0.499, 0.249	.495	0
Relaxation	−0.179	−0.434, 0.077	.160	16.0 %
Social Skills Training	−0.032	−0.307, 0.243	.809	0
Homework	0.021	−0.267, 0.308	.882	0
Therapist Involvement	0.039	−0.293, 0.372	.809	0
Parent Involvement	−0.111	−0.433, 0.211	.480	0
Duration	−0.013	−0.046, 0.019	.407	0
Intensity	0.013	−0.034, 0.059	.585	0

*k* = number of comparisons. ${\hat{\beta }}_{2}$ = partial regression coefficient of the moderator. *p* = *p*-value of the statistical testing for ${\hat{\beta }}_{2}$. $\Delta R^{2}$: increment in the estimate of pseudo-$R^{2}$ (taking the model with type of comparator only as a reference).

As for anxiety, Table 6 shows the results from meta-regression analyses. IDPT interventions including cognitive restructuring were significantly more effective (*b* = 0.292, 95 % CI = 0.028 to 0.555), accounting for an additional 53 % of variance among effect sizes. None of the other moderators yielded statistically significant results. Last, funnel plots – see Figure s3 – showed little evidence of asymmetry among effect sizes comparing IDPT to active control (*p* = .20) or to inactive control (*p* = .11).

**Table 6:** Results of the mixed-effects meta-regression models applied on effect sizes for anxiety (*k* = 23).

Moderator	${\hat{\beta }}_{2}$	95 % CI for ${\hat{\beta }}_{2}$	*p*	$\Delta R^{2}$
% Clinical Population	0.002	−0.000, 0.005	.090	14.6 %
Depressive	−0.204	−0.500, 0.092	.168	11.7 %
Anxious	0.216	−0.138, 0.570	.220	1.48 %
Focus	−0.047	−0.354, 0.260	.754	0
Emotion Count	0.081	−0.239, 0.400	.605	0
Emotion Regulation	0.031	−0.252, 0.314	.821	0
Problem Solving	−0.217	−0.493, 0.058	.116	6.71 %
Cognitive Restructuring	0.292	0.028, 0.555	.032	53 %
Behavioural Activation	−0.120	−0.409, 0.169	.400	0
Gradual Exposure	0.238	−0.098, 0.575	.155	10.8 %
Relaxation	0.002	−0.279, 0.284	.986	0
Social Skills Training	0.076	−0.200, 0.353	.573	0
Homework	−0.067	−0.375, 0.241	.655	0
Therapist Involvement	0.113	−0.222, 0.448	.492	0
Parent Involvement	0.067	−0.241, 0.375	.657	0
Duration	0.031	−0.002, 0.065	.064	27.7 %
Intensity	0.022	−0.016, 0.061	.246	10.2 %

*k* = number of comparisons. ${\hat{\beta }}_{2}$ = partial regression coefficient of the moderator. *p* = *p*-value of the statistical testing for ${\hat{\beta }}_{2}$. $\Delta R^{2}$: increment in the estimate of pseudo-$R^{2}$ (taking the model with type of comparator only as a reference).

## Discussion and conclusions

The purpose of this study was to investigate the efficacy of IDPT interventions to improve internalizing symptomatology of anxiety and depression in a child and adolescent population with clinical or sub-clinical symptoms, and to estimate the efficacy of IDPT compared to different comparison groups.

To the best of our knowledge, this is the first network meta-analysis of IDPT interventions to reduce clinical or sub-clinical internalizing symptoms in children and adolescents. We found sufficient evidence to conclude that IDPT interventions to reduce depression are effective in the child and adolescent population when compared with active and inactive control groups. However, we did not find enough evidence to conclude that these interventions had a more beneficial effect on depression when compared with other types of interventions such as “Attention Bias Modification Training”, “Hybrid Therapy”, “Social Cognitive Theory” or “Face-to-face Therapy”. These results must be interpreted cautiously due to the low number of studies that compared IDPT with these types of interventions. Similar results were found for the anxiety variable, with a more beneficial effect being observed when IDPT interventions were compared with active or inactive control groups, but insufficient evidence was found to affirm the superiority of these interventions over others like “Attention Bias Modification Training” or “Face-to-face Therapy”. Although we found superiority of psychodynamic-type treatments over IDPT for anxiety, these results must be interpreted with caution, as only one study reported this comparison.

Residual heterogeneity was found in our results, which led us to analyze characteristics of studies which might explain at least part of the heterogeneity of effect sizes, focusing on comparisons of IDPT interventions with active and inactive control groups. No statistically significant moderators of effect sizes were found for depression, whereas for anxiety the cognitive restructuring component was shown to be a significant moderator of effect sizes. We believe that due to high comorbidity between anxiety and depression (Baartmans et al., 2022; Davies et al., 2023; De Vries et al., 2022), it is likely that the cognitive restructuring component also modulates effect sizes for depression. Although no significant moderators were found for the latter, evidence of publication bias found when IDPT interventions for depression were compared with an active control group is likely to have influenced these results. In addition, other studies on the technological and delivery characteristics of IDPT programs observed that when elements of depression-based cognitive behavioral therapy for adolescents were incorporated into the treatment, outcomes improved regarding depression (Wozney et al., 2017) and anxiety (Rooksby et al., 2015).

IDPT interventions for internalizing problems have been extensively studied. In ours, moderate and significant effect sizes have been observed (Cohen, 1988) for anxiety and depression when compared to active or inactive control groups. Our results are in line with the findings of other studies (Buttazzoni et al., 2021; Ye et al., 2014), which suggest that IDPT interventions produce moderate to low effects in reducing depression and anxiety symptomatology, respectively. However, other papers find large effect sizes when examining the effectiveness of IPDT versus control groups for anxiety and depression (Karyotaki et al., 2018; Valimaki et al., 2017). Nonetheless, these papers are based on a small number of comparative groups, in contrast to the groups included in our review (*k* = 71), they mix a child and adolescent population with a young adult population, and/or have been conducted with an adult population.

The past experience of confinement and the current circumstances of the need to maintain complementary intervention programs or supporting face-to-face therapies make it convenient to implement telehealth tools (videoconferences, online forums, apps, e-mails, etc.) to address negative effects in the mental health of the population during the pandemic (Zhou et al., 2020), and which have continued to grow in the post-pandemic era. Internet-based approaches could help to reach target groups who otherwise cannot access conventional treatment or do not receive it at the required time or intensity (Andersson & Cuijpers, 2009).

Our study had some limitations. Firstly, our results focused on symptom reduction and not on reduction of clinical diagnoses, which might also be of interest to those responsible for the application of the IDPT in the child and adolescent population. Second, we did not have enough data to examine intervention effects separately for children and adolescents, and we recognize that the transitivity assumption might be compromised for some interventions. Third, most IDPT interventions were compared with active and inactive control groups, with only a small number of papers comparing these interventions with other active treatments, hence the effects for these comparisons could not be precisely estimated. Last, the lack of registration of the review protocol in a recognized registry could have resulted in a risk of bias in our study affecting its usefulness for clinical and research decision making.

Regarding the strengths of our study, firstly, it is worth note that we conducted extensive searches through five different databases and covering a wide range of years, aiming to find all relevant studies. Second, studies were screened and coded by two independent reviewers, following an updated coding manual, which promotes the objectivity and rigor of the study and helps to overcome the limitations associated with not including the review protocol in a registry. Third, studies came from four different continents (Europe, North America, Oceania and Asia), which may facilitate the generalization of our results to different geographic locations. Fourth, most papers included in this review met numerous criteria of methodological quality, samples were homogeneous in terms of age and problems, and treatments analyzed were specifically developed for clinical and sub-clinical populations, thus overcoming most of the problems found in other meta-analyses (Rooksby et al., 2015; Ye et al., 2014). Finally, the most recent and sophisticated synthesis methods (i.e., network meta-analysis) were applied in this systematic review.

The main practical and research implication of this meta-analysis is that our work may help other IDPT developers to analyze which components work best in this type of intervention, and the cognitive restructuring component has been shown to be most relevant. In addition, most programs contained a psychoeducation component and an emotion acknowledgement component, so that future developments of IDPT for this type of population should consider incorporating these components into treatment programs. Providing online information based on interactive elements, such as games or practical exercises, about the symptoms, causes and mechanisms of anxiety and depression can help children and adolescents understand their emotional and cognitive experiences, and teaching effective strategies for managing symptoms of anxiety and depression, such as cognitive restructuring techniques, can be instrumental in improving the emotional well-being of children and adolescents. IDPTs have been shown to be more effective than control groups, both active and inactive, making them a therapy of choice in the absence of other types of interventions, facilitating universal accessibility to psychological therapy, reducing the stigma associated with seeking help for mental health problems, making the timing of interventions more flexible so that they can be tailored to the individual needs of children and adolescents, and increasing the audience for interventions compared to traditional face-to-face services.

Finally, we conclude that IDPT interventions to reduce internalizing symptoms of anxiety and depression in children and adolescents with clinical or sub-clinical symptomatology are effective and are a highly recommended option, especially when children and adolescents cannot receive conventional treatment for different reasons (such as living in rural areas, long waiting lists for access to specialized professionals, etc.). Nevertheless, more studies are needed to provide evidence on the efficacy of IDPT versus other types of interventions such as face-to-face therapy, since the small number of studies comparing these interventions is not sufficient to establish solid conclusions as to which type of intervention format is more effective.

## Funding

This study was funded by Agencia Estatal de Investigación (Government of Spain). PID2019-104033GA-I00 /MCIN/AEI/10.13039/ 50110 00110 33, PID2019-104080GB-I00/MCIN/AEI/10.13039/501100011033, and by the Region of Murcia (Spain) through the Regional Program for the Promotion of Scientific and Technical Research of Excellence (Action Plan 2022) of the Seneca Foundation - Science and Technology Agency of the Region of Murcia (grant no. 22064/PI/22).

## Declaration of competing interest

The authors declare that they have no known competing financial interests or personal relationships that could have appeared to influence the work reported in this paper.

## Appendix Supplementary materials

Image, application 1
